# On normative judgments and ethics

**DOI:** 10.1186/s12910-016-0155-8

**Published:** 2016-11-22

**Authors:** Ognjen Arandjelović

**Affiliations:** School of Computer Science, University of St Andrews, North Haugh, KY16 9SX St Andrews, UK

**Keywords:** Enhancement, Treatment, Natural, Non-natural

## Abstract

Recent rapid technological and medical advance has more than ever before brought to the fore a spectrum of problems broadly categorized under the umbrella of ‘ethics of human enhancement’. Some of the most contentious issues are typified well by the arguments put forward in a recent article on human cognitive enhancement authored by Garasic and Lavazza. Herein I analyse some of the assumptions made in their work and highlight important flaws. In particular I address the problems associated with the distinction between ‘treatment’ and ‘enhancement’, and ‘natural’ vs. ‘non-natural’ therapies.

It is with much interest that I read the article entitled “Moral and social reasons to acknowledge the use of cognitive enhancers in competitive-selective contexts” authored by Garasic and Lavazza, and recently published in BMC Medical Ethics [1]. The topic addressed in the aforementioned article is highly relevant and intellectually interesting, so I very much welcome all contributions to the surrounding debate. Moreover I found the authors’ thoughts and arguments insightful, and am in agreement with many of their conclusions.

Having said the above, I am compelled to highlight a number of important premises underlying the arguments of Garasic and Lavazza, which though appealing at first sight fail to withstand deeper scrutiny. For the sake of succinctness I will focus on a few which I find to be the most pervasive in the existing literature, or which I consider to be of greatest conceptual importance for the correct framing of the discussion.

Enhancement vs. treatment I would like to begin with the distinction that Garasic and Lavazza attempt to draw between the use of various means to achieve an enhancement of a human ability (in this case the authors are specifically interested in cognitive abilities but the nature of the argument remains unaltered even if this constraint is abandoned) as opposed to what is usually termed as a treatment. The former is supposed to be understood as effecting an improvement to an ability initially functioning within the suitable normal range, or to quote Garasic and Lavazza (remembering that their focus is on pharmaceutical agents and cognitive performance):

“The substances most commonly included in the set of PCE are drugs used off-label by healthy people who do not have specific deficits but want to improve their standards of intellectual and cognitive performance…”,

whereas the latter is taken to be a remedy for sub-normal functioning (or indeed, a full loss of a specific function) thereby treating what can be considered an abnormality (e.g. a disability, disfunction, or disorder). The same distinction has been made previously by a number of scholars [2–4], and though it may appeal to our intuitive sensibilities I believe that a more rigorous consideration and analysis of the distinction, made by several authors in the past [5, 6], reveals its lack of a solid underpinning principle.

Humans exhibit variation in nearly if not literally every characteristic worthy of consideration as well as perhaps more pertinently, in the potential for the development of a particular characteristic, be it height [7], muscular strength [8], memory [9], sense of spatial orientation [10], general intelligence [11], or any one of a plethora of other possible examples. What range within this spectrum we deem as normal is in principle arbitrary { individuals (in this case human but also more generally of any life form) with a well adapted or poorly adapted particular characteristic are expected to be found in nature, as the theory of natural selection would predict. There is no fundamental reason why any of them would be considered ‘abnormal’ or ‘not normal’. They may be desirable or undesirable, from the point of the view of the individual himself, another individual, or (more contentiously) even the society, but the realm of these descriptions is different from that occupied by claims of ‘normality’ or lack thereof.

Even if we accept a certain delineation between normal and sub-normal, it is not at all clear when treatment would become enhancement. Should ‘treatment’ merely take a certain function to the lowest end of the ‘normal’ range? Why or why not instead the mean of the ‘normal’ range or indeed its upper limit? Why would the use of a certain agent not be considered as enhancement if the person’s characteristic of interest is marginally sub-normal but as enhancement if the person is marginally above the sub-normal limit? What moral principle impels us to treat a climb on the ladder of competitiveness (recall that the authors’ focus is on competitive contexts) of the former individual differently than that of the latter?

The aforementioned variation in human characteristics, of course, also exhibits temporal variation [9, 12]. It is not in the least surprising that, for example, as a person ages there is an associated deterioration in a number of physical and mental functions. Should the boundaries of ‘normal’ thus vary accordingly? Does that for example mean that the hormone replacement therapy (HRT) administered to a menopausal woman would fall under the umbrella of ‘enhancement’ even if it results in higher quality of life?

In brief, the authors’ argument is in effect underlain by an essentialist view whereby there is some idealized ‘normally functioning’ human. However this stance does not fit well our scientific understanding of the biological reality, the genotypic and phenotypic diversity fundamental to the principle of natural selection, or indeed ethical principles.

Natural vs. non-natural Another distinction that the authors attempt to make early in their article is that of ‘natural’ and ‘non-natural’ enhancements^1^:

“It seems reasonable to stress that there are substantial differences between the natural enhancers we just mentioned and the newer ones…”

The authors do not explain clearly what they mean by ‘natural’ in this context. Presumably what they refer to are substances which occur in nature without human intervention (their examples of caffeine, nicotine, and khat, support this interpretation). Even if we accept this understanding of the term ‘natural’ and neglect the observation that these ‘natural’ substances are often sold and consumed in concentrations and forms very much unlike those found in nature (e.g. caffeine is readily bought as 99.9 % pure powder [13]), as well as consumed in a fashion which their natural forms would not readily allow (e.g. transdermally [14] or using nasal sprays [15]), I fail to see how this definition is morally relevant. Indeed the authors seem to be obliquely aware of this as suggested by their attempt to elaborate the issue using a non sequitur whereby the focus is shifted to the magnitude of effect:

“…in other words, the ‘peak’ of the performance is not comparable to that sought through the use of methylphenidate and Modafinil, for instance [15{17].”

Notwithstanding the examples given there is no fundamental reason why a ‘natural’ substance would not have a substantial effect. If effectiveness is indeed what the authors are interested in, surely it is in fact not reasonable to make a distinction between agents based on whether or not they are ‘natural’ but rather based on their inherent properties. However if this approach is adopted similar problems to those highlighted previously in “Enhancement vs. treatment” arise.

Continuing with an attempt to exclude some agents from their consideration the authors shortly thereafter make another shift in their position summarized by the following:

“The substances that are currently most widely used (and deemed to be the most effective) are those marketed for the treatment of neurodegenerative disorders, ADHD and narcolepsy [38{40]. Thus, caffeine and nicotine, as well as other forms of enhancement, are excluded from the domain of PCE.”

The reasoning in this excerpt is oddly singular. The authors seem to shift their position from the consideration of whether specific agents (in this case caffeine and nicotine) are found in nature or how effective they are, to how they are marketed. Even more so than in the previous cases it is difficult to see what relevance in the context of the present discussion the authors find in this.

Lastly, though I will not elaborate on this point, given the nature of Garasic and Lavazza’s article I consider their unquestioned and unqualified adoption of the term ‘abuse’ (in “…Federal Substance Abuse and Mental Health Services Administration affirms that roughly 137,000 American college students start abusing prescription stimulants each year.”) an unfortunate oversight.

## Footnotes

1 Though it is reasonable to assume that the authors are trying to contrast ‘natural’ with ‘synthetic’ since they do not actually use the latter term explicitly (nor any other in its place), in an e ort to avoid the perception of making a straw man argument I use the adjective ‘non-natural’ as an ipso facto class that includes all agents which are not ‘natural’ and only those.
