# Supplementary material for: On normative judgments and ethics
Source: BMC Med Ethics. 2016 Nov 22;17:75. doi: 10.1186/s12910-016-0155-8 (PMC5120522; doi:10.1186/s12910-016-0155-8)
Supplement: Related texts

# Reply: Response to: “On normative judgments and ethics”

**Authors:** Mirko Daniel Garasic, Andrea Lavazza

**Affiliations:** 2 Edmond J. Safra Center for Ethics, Tel Aviv University, Tel Aviv, Israel; 3 Centro Universitario Internazionale, Arezzo, Italy

We would like to thank Arandjelović for having engaged with our article and having praised some of its content. Here we will try to reply to some of the concerns raised by him hoping to clarify further some of the aspects deemed unclear.

## Enhancement vs. treatment

Regarding the often discussed issue of enhancement vs. treatment, we agree that there is no “ontological” divide between normality and abnormality, and that “humans exhibit variation in nearly if not literally every characteristic worthy of consideration”. However, in our opinion, this shareable observation is not enough to completely invalidate this distinction, which can be drawn from objective data and involves a conventional and/or normative component. Take, for example, the main neuropsychological tests used for cognitive and specifically executive functions. These tests, which have been standardized and validated by the scientific community well before the debate on enhancement, assume that there is some degree of variability in cognitive performance, but also set the minimum thresholds, under which it is agreed, and not arbitrarily, that the subject has a deficit. If this is the case, it is understood that the subject cannot perform adequately - or safely for herself and for others - certain tasks requiring cognitive and executive abilities at a higher level than the established minimum.

For instance, if someone lacks sufficient attention and reflexes, they cannot be given a driving license, and an intervention, pharmacological or otherwise, to restore sufficient testable levels should be considered a treatment. In contrast, if a subject falls within the normal range and, hoping to become a Formula 1 driver, takes massive doses of Ritalin, then this is enhancement. Of course, there may be more nuanced situations, and the enhancement vs. treatment question can never be settled for good. Still, the distinction is valid and can be useful. It obviously is not absolute, nor is it solely based on “our scientific understanding of the biological reality,” but it can still be used to ground a thought out moral evaluation and a pondered practical application in social and political choices.

A very interesting aspect, which we have not dealt with as it lies outside the scope of our article, is the question raised by Arandjielović about temporal variations in human characteristics. How to evaluate interventions on older people, who are facing a progressive physiological deterioration of their cognitive skills? When, hopefully soon, it will become possible to treat Alzheimer’s patients who, in the advanced stage of the disease, have lost almost all of their memory, will this be considered a treatment? And what about restoring the memory of a healthy nonagenarian to levels typical of a forty-year-old? Will that be considered enhancement? Deciding whether this distinction makes sense and can be drawn will obviously have strong practical implications, linked, for example, to the interventions that public health systems will be required to provide, for free or charged to the patient.

## Natural and non-natural

Arandjelović is correct in underlining that we did not fully specify the meaning of natural, so we will take this occasion to expand on this point.

We are aware that the definition of ‘natural’ -or normal- 2 can easily become controversial and whatever the limits one assesses to be appropriate, they can be questioned. Yet, we opted for the use of one distinction.

The commentator suggests synthetic as a possible alternative to define all the substances not considered natural -giving relevance to the fact of those being created in a laboratory. Even if we do not use the term directly in our article, the natural/synthetic distinction holds –albeit it does encapsulate all the meaning of our definition.

To clarify better –and address another concern of Arandjelović- we must point out that the definition is morally relevant for the importance it conveys to the accessibility that a given substance has in a given society -exemplifying at the same time the ‘historical roots’ of a certain (cognitive) enhancer -with the resulting acceptance and distribution (the use and problems associated with Khat is linked to this for instance), as well as a gradual biological adaptation of our body to the effects of the substance.

In line with the ideology at the core of human enhancement (to which supporters of “more effective” cognitive enhancers refer to more or less directly), one of the objectives of any enhancement is to jump as many as possible of the ‘natural phases’ of development occurred in the course of human evolution. So, even if caffeine is consumed as a result of an “artificial” process not present in nature, the characteristics of the substance itself have not been modified much. In addition, the cultural development of having implemented coffee in our lives might be ascribable also to its taste, not only the way it affects our performances (the same could be said for cacao in relation to our emotional cravings for instance). On the other hand, amphetamines of various kinds have been created in laboratories with the only intention of amplifying our concentration, focus, resistance to tiredness and so on. That is what, in our account, makes a difference. And the relevance of such a distinction is exemplified by the stronger effects that all the PCE defined by us as non-natural have.

As for the “unfortunate oversight” of having used the term ‘abuse’ in relation to the use of PCE by college students not in need of such prescription drugs, we might concede that ‘misuse’ might be a slightly more neutral term –though ‘abuse’ remains obviously more directly linked to the very authority we cite the data from.
